# Expression of Multiple Exogenous Insect Resistance and Salt Tolerance Genes in *Populus nigra* L.

**DOI:** 10.3389/fpls.2020.01123

**Published:** 2020-07-24

**Authors:** Xinglu Zhou, Yan Dong, Qi Zhang, Dandan Xiao, Minsheng Yang, Jinmao Wang

**Affiliations:** ^1^ Institute of Forest Biotechnology, Forestry College, Agricultural University of Hebei, Baoding, China; ^2^ Hebei Key Laboratory for Tree Genetic Resources and Forest Protection, Baoding, China; ^3^ Institute of Coastal Agriculture, Hebei Academy of Agriculture and Forestry Sciences, Shijiazhuang, China

**Keywords:** multi-resistance gene, multigenic vector, *Populus nigra* L., insect resistance, salt resistance

## Abstract

Four exogenous genes, *Cry3A*, *Cry1Ac, mtlD,* and *BADH*, were inserted into the p1870 vector to obtain multigenic transgenic *Populus nigra* L. with improved insect resistance and salt tolerance. During vector construction, different promoters were used for each gene, the AtADH 5′-UTR enhancer was added between the *Cry1Ac* promoter and the target gene, and the matrix attachment region (MAR, GenBank: U67919.1) structure was added at both ends of the vector. It was then successfully transferred into the genome of European black poplar by Agrobacterium-mediated leaf disk transformation, and a total of 28 transgenic lines were obtained by kanamycin screening. Five events with the highest insect resistance were selected based on preliminary tests: nos. 1, 7, 9, 12, and 17. PCR, real-time PCR, and enzyme-linked immunosorbent assays (ELISA) were used to detect the expression of exogenous genes and to analyze the Bt protein toxin levels in transgenic lines from June to October. PCR results showed that all four genes were successfully introduced into the five selected lines. Fluorescence quantitative PCR showed no significant differences in the transcript abundance of the four exogenous genes between different lines. A Bt protein toxin assay showed that the Cry3A protein toxin content was significantly higher than the Cry1Ac protein toxin content by approximately three orders of magnitude. Levels of the two toxins were negatively correlated. Over the course of the growing season, Cry1Ac content raised and varied between 0.46 and 18.41 ng·g^−1^. Cry3A content decreased over the same time period and varied between 2642.75 and 15775.22 ng·g^−1^. Indoor insect feeding assay showed that the transgenic lines had high insect resistance, with mortality rates of 1–2-year-old *Hyphantria cunea* larvae reaching more than 80%, and those of *Plagiodera versicolora* larvae and nymphs reaching 100%. No. 17 and no. 12 lines had better insect resistance to *Lepidoptera* and *Coleoptera* pests. There was no clear improvement in salt tolerance of the transgenic lines, but comprehensive evaluation of 11 salt tolerance indicators showed that lines no. 17 and no. 7 had certain degrees of salt tolerance.

## Introduction 

Pests and soil salinization are key factors that restrict forestry production and development (Zhu, 2001; Dennis and Andrea, 2012; Li and Li, 2018). Genetic improvements based on transgenic technology can play an important role in the prevention and control of diseases and pests and permit efficient use of land with saline and alkaline soils. Cultivation of insect-resistant and salt-tolerant genetically modified superior varieties is an important development goal (Fladung et al., 2013; Movahedi et al., 2015; Yang et al., 2016), and use of the microbial insect-resistance gene *Bt* for the development of transgenic plants has received significant attention (Hou and Chen, 2000). Compared with other insect-resistance proteins, the protein toxins encoded by *Bt* have the strongest insect-resistance ability at equivalent expression levels, and, after modified transformation, the *Bt* gene has greatly improved resistance plant insect (Schuler et al., 1998; Génissel et al., 2003). In transgenic trees, the *Cry1* gene has conferred specific resistance to *Lepidoptera* pests with high efficiency, whereas the *Cry3* gene has conferred specific resistance to *Coleoptera* pests with high efficiency (Yang et al., 2008).

In recent years, salt tolerance genes such as 1-phosphomannitol dehydrogenase (*mtlD*) and betaine aldehyde dehydrogenase (*BADH*) have become the focus of research on the genetic engineering of stress tolerance (Zhu and Wang, 2015). The product of the *BADH* gene pathway, betaine, cannot be further metabolized to any great extent after synthesis. It therefore serves as a permanent or semi-permanent osmotic regulator and is considered as one of the most promising osmotic protectants (Shu et al., 1997). It has received increasing attention in salt and drought stress research, and has been used to generate transgenic plants with increased drought resistance and salt tolerance.

Genetic engineering of transgenic trees has mainly involved changes to specific traits through transformation with one or two genes (Liu et al., 2007; Naqvi et al., 2010). As reports on pest tolerance and low protein toxicities have increased in number, multigene transformation (two or more toxin-encoding genes), and combinations of the *Bt* gene with other genes, have been used to create cumulative insecticidal effects. The transfer of two or more different resistance genes into the same plant and the simultaneous improvement of multiple plant traits have become the focus of transgenic research (Bates et al., 2005; Li and Zhu, 2005; Christou et al., 2006; Wang et al., 2010). Multigene transformation can introduce multiple genes, such as insect resistance and salt tolerance genes, into the same plant genome, altering multiple traits at the same time and improving the comprehensive resilience of plants(Lian et al., 2008; Sun et al., 2011). However, multigene genetic transformation still faces a series of technical challenges, such as exogenous gene silencing, multigene interaction, and efficient expression (Jin et al., 2015; Ren et al., 2015). It is particularly important to explore the stable existence and efficient expression of exogenous genes during multigenic transformation (Ren et al., 2017). The construction characteristics of different vectors play an important role in promoting the expression of exogenous genes (Dong et al., 2015). Different promoters can be used for each gene to avoid gene silencing by excessive promoter re-use (Wang H. H. et al., 2008).

Plant genetic engineering makes use of multiple promoters, among which the CaMV35S promoter is particularly strong. Many other strong promoters can replace CaMV35S in monocots, but expression of promoters other than CaMV35S in dicots is relatively weak (Schledzewski and Mendel, 1994; Yuan et al., 2000; Zhang et al., 2005; Nie et al., 2008). The MAR structure can reduce the influence of plant regulatory mechanisms on target gene expression, enhance the expression of exogenous target genes, and improve the expression ability of exogenous genes (Allen et al., 1996; Hily et al., 2009). When foreign genes have close homologs in the genome, they may interfere with one another and cause gene interactions. The insertion of enhancers between foreign genes and promoters is an important means for improving the expression of target genes (Stief et al., 1989).

In this study, two insect-resistance genes, *Cry1Ac* (GenBank AF148644.1) and *Cry3A* (GenBank M84650.1), as well as two salt-tolerance genes, *mtlD* (Gene ID 948117) and *BADH* (GenBank DQ497233.1), were inserted into the same multi-gene expression vector. The promoters of the exogenous genes were optimized, and multiple regulatory elements were added. Using single carrier co-transformation, the resulting construct was transferred to superior clones of European black poplar. Molecular and biological analyses were performed on the transgenic plants, and the expression of the four exogenous genes was analyzed to provide a reference for efficient exogenous gene expression following multi-gene transformation in poplar.

## Materials and Methods

### Materials

#### Strains and Vectors

**Figure 1:**
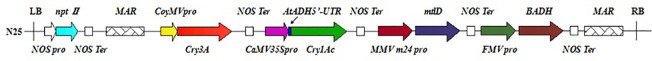
Construction of plant transformation vector with multiple insect-resistant and salt-tolerant genes. N25, the code name of the process bacterium formed after vector transfer to agrobacterium; LB, vector left boundary; NOS pro, promoter of neomycin phosphotransferase gene; *nptII*, neomycin phosphotransferase gene; NOS Ter, terminator of neomycin phosphotransferase gene; MAR, the molecular structure of the matrix attachment region of tobacco; CoyMV pro, commelina yellow mottle virus promoter; *Cry3A*, bacillus thuringiensis insecticidal crystal protein gene; CaMV35S, cauliflower mosaic virus CaMV35S promoter; AtADH5’-UTR, translation enhancer; *Cry1Ac*, bacillus thuringiensis insecticidal crystal protein gene; MMV m24 pro, mirabilis mosaic virus promoter; *mtlD*, 1-phosphomannitol dehydrogenase gene; FMV pro, figwort mosaic virus promoter; *BADH*, betaine aldehyde dehydrogenase gene; RB, right boundary of vector.

The *Escherichia coli* strain DH10B, and the *Agrobacterium tumefaciens* strain GV3101 (rifampicin resistant) were used for genetic transformation. The plant expression vector was p1780, and different promoters were used for each gene. The AtADH 5′-UTR translation enhancer was added between the *Cry1Ac* promoter and the target gene, and the 1168 bp MAR structure was added at both ends of the four target genes. The complete structure is shown in **Figure 1**.

#### Plant Materials

The plant material used for genetic transformation was eu-1, a superior clone of *P. nigra.* Non-transgenic eu-1 (CK) was used as control. Eu-1 was a wild-type plant provided by Professor Hu at Research Institute of Forestry, Chinese Academy of Forestry. The transgenic lines and control plants were planted in a randomized block design under the same conditions at the same nursery site.

#### Test Insects

*Hyphantria cunea* belongs to the order *Lepidoptera* in the *Arctiidae*. Eggs of *H. cunea* were provided by Professor Chen at Beijing Forestry University. *Plagiodera versicolora* belongs to the order *Coleoptera* in the *Chrysomelidae*. Adults and eggs were collected from Baoding in Hebei province.

### Methods

#### Genetic Transformation With the Agrobacterium-Mediated Leaf Disk Method

Genetic transformation was performed as described in Liu et al. (2016). Multiple foreign genes were integrated simultaneously into the genome of *P. nigra* using Agrobacterium-mediated leaf-disk transformation. Transgenic plantlets were obtained by kanamycin screening, then acclimated and transplanted to a nursery site for reproduction.

#### PCR Detection of Transgenic *P. nigra*

In early July during the plant growth period, fully expanded young leaves of all plant lines and controls were collected from the field. The CTAB method was used to extract DNA from three biological replicate plants of each line. Specific primers were designed according to each exogenous gene sequence. PCR was used to detect foreign genes and to determine whether exogenous genes were integrated into the eu-1 genome of *P. nigra*. All PCR primers are shown in **Table 1**. The 20 μl reaction system contained 2 μl 10×PCR Buffer, 2 μl dNTP, 1 μl forward primer, 1 μl reverse primer, 0.2 μl rTaq DNA polymerase, 1 μl DNA template, and 12.8 μl ddH_2_O. The PCR reaction conditions for each exogenous gene were as follows:

**Table 1:** PCR primers for exogenous genes.

Exogenous gene	Strip size (bp)	Forward primer	Reverse primer
*Cry1Ac*	546	ATGGATAACAATCCGAACATCA	CCACCTTTGTCCAAACACTGAA
*Cry3A*	667	CACTGTTCCCACTGTACGATGT	ATGTTGAAGAAGTCCACGCTCT
*mtlD*	409	ACCGCCTTCGGCAACTAAC	TGGCTGACGGCCTACGCGCTCTACA
*BADH*	507	TGGTGCTCATCGTGCTAAAT	CTCCCAGTAAATGCTACCTTGT

*Cry1Ac* and *Cry3A*: predenaturation at 95°C for 6 min, denatured at 94°C for 50 s, annealed at 51°C for 55 s, extended at 72°C for 55 s (30 cycles), extended at 72°C for 5 min. *MtlD*: predenaturation at 94°C for 5 min, denaturation at 94°C for 30 s, annealing at 51°C for 40 s, extension at 72°C;f or 2 min (30 cycles), extension at 72°C for 15 min. *BADH*: predenaturation at 95°C for 6 min, denatured at 94°C for 50 s, annealed at 51°C for 55 s, extended at 72°C for 55 s (30 cycles), extended at 72°C for 5 min.

#### Fluorescence Quantitative PCR Detection

In early July three biological replicate samples of fully expanded young leaves from all plants and controls were collected from the field, frozen in liquid nitrogen, and stored at −80°C. Total RNA was extracted according to the instructions of the EASYE×PLUS plant RNA kit (Saylor Biotechnology). cDNA was obtained by reverse transcription using a kit from the Beijing Adlai company. cDNA was used as the template, and the 2×Sybr Green qPCR Mix was used for quantitative fluorescence PCR. Using plasmid DNA that contained foreign genes of known fragment size as the standard, fluorescence quantitative PCR was used to generate a standard curve by 10X serial dilution

Real-time fluorescent quantitative PCR primers were designed based on the full sequence information of the exogenous genes, and all fluorescence quantitative PCR primers are shown in **Table 2**. Based on the number of cycles (Ct value) required for the fluorescence signal in each PCR reaction to reach the set domain value, the abundance of each cDNA was calculated using the standard curve. PCR reaction conditions for the *Bt* genes and the salt tolerance genes were slightly different. *Cry1Ac*, *Cry3A*: predenaturation at 95°C for 5 min, denaturation at 95°C for 30 s, annealed at 60°C for 30 s, extension at 72°C for 30 s (40 cycles), extension at 72°C for 2 min; *mtlD* and *BADH*: predenaturation at 95°C for 5 min, denaturation at 95°C for 30 s, annealing at 62°C for 30 s, extension at 72°C for 30 s (40 cycles), and extension at 72°C for 2 min.

**Table 2:** Fluorescence quantitative PCR detection primers for target genes.

Target gene	Strip size(bp)	Forward primer	Reverse primer
*Cry1Ac*	167	GAATTTTTGGTCCCTCTCAAT	AGGATCTGCTTCCCACTCTCT
*Cry3A*	203	TGGGGATACGAGAAGGAGGAT	AGTGGGAACAGTGCGATGAGA
*mtlD*	146	GCCGAACATCCCAGGCATGG	CGTCGAGAATCGCGTCACGA
*BADH*	145	CCCAATTCCTGCTCGTCAACTCT	CACTGCAACCTCCACATCCTCTG

### ELISA Assay of Transgenic Lines and Spatiotemporal Expression of the Bt Protein Toxin

Transgenic and control plants were planted under the same conditions at the same nursery site. Once a month from June to October, three biological replicate samples of similar leaves were collected from all transgenic lines and controls and stored in an ultra-low temperature freezer. Bt protein toxin contents were measured by ELISA using a BioRad 550 microplate reader, following the directions of the Cry1Ac and Cry3A toxin protein kits (Agdia, USA). The absorbance value and concentration of the standard sample were used to develop a standard curve, and the toxin content of each sample (g per g fresh leaf weight) was calculated based on its absorbance and fresh weight.

### Insect Resistance Assays

The effect of *Cry1Ac* was measured using *H. cunea* 1–6 instar larva, and the effect of *Cry3A* was measured using *P. versicolora* larvae, nymphs, and adults. Fresh leaves from transgenic and control seedlings were collected from the nursery and their petioles were inserted into moist floral clay. Insects were placed evenly onto the leaves, and the leaves and insects were placed into wide-mouthed glass bottles with a diameter of 6.5 cm and a height of 8 cm. Bottles were covered with wet gauze, sealed with rubber bands, and placed inside plastic bags. Thirty insects were used in each feeding assay, and all assays were replicated three times. The number of dead insects was recorded each day until it remained stable or all insects had died. Mortality was calculated as:

Mortality = number of dead insects/total number of insects × 100%

### Determination of Salt Tolerance Indicators

Transgenic plant lines and control hardwood cuttings were placed in soil-filled containers with a diameter of 40 cm and a height of 30 cm. After 30 d of growth, a salt tolerance assay was performed using two treatments: water or 3‰ NaCl. applied to the soil surface. Water or salt solution was re-applied every 4 d for 20 d, and each treatment was replicated three times.

Eleven indicators were measured for each transgenic line, including plant height, ground diameter, and leaf conductivity. A spectrophotometer was used to measure absorbance at multiple wavelengths, and the contents of chlorophyll a, chlorophyll b, and carotenoids in leaves were calculated. Superoxide dismutase (SOD) activity, malondialdehyde (MDA) activity, soluble protein content, and additional indices were also measured under salt stress. Plant mannitol and betaine kits were used to measure the contents of *mtlD* and *BADH* pathway products. The fuzzy mathematical membership function method (Liu et al., 2010) was used to quantitatively transform the eleven indicators, and the average membership degree of each indicator was used as the comprehensive identification standard to compare salt tolerance among the transgenic lines. The following formula was used for the measured indicators:

$$U(X_{i})=(X_{ij}-\;X{\;}_{\min\;i})/(X{\;}_{\max\;i}-X{\;}_{\min\;i}),\Delta =1/n\Sigma U(X_{i})$$

where *X_ij_*represents the *i*th determination index of the *j*th clone U(Xi)∈[0,1], and Δ is comprehensive evaluation result for each index based on *n* measurements.

Relative conductivity was calculated using an inverse membership function:

$$U(X_{i})=1-(X_{ij}-X{\;}_{\min\;i})/(X{\;}_{\max\;i}-X{\;}_{\min\;i}),\Delta =n\Sigma U(X_{i})$$

### Statistical Analyses

All statistical analyses were performed using Microsoft Excel 2003 software, the results are means of three biological replicates of each line, error bars represent the standard deviation of the mean. SPSS 22.0 software was used to analyze the data of one-way analysis of variance (ANOVA), followed by Duncan’s multiple range test, the level of significance was set at *p* < 0.05 for all treatments (Duncan, 1995). All the graphs were created using Origin Pro 9.0.

## Results and Analysis

### Preliminary Screening of Transgenic Lines for Insect Resistance

**Figure 2:**
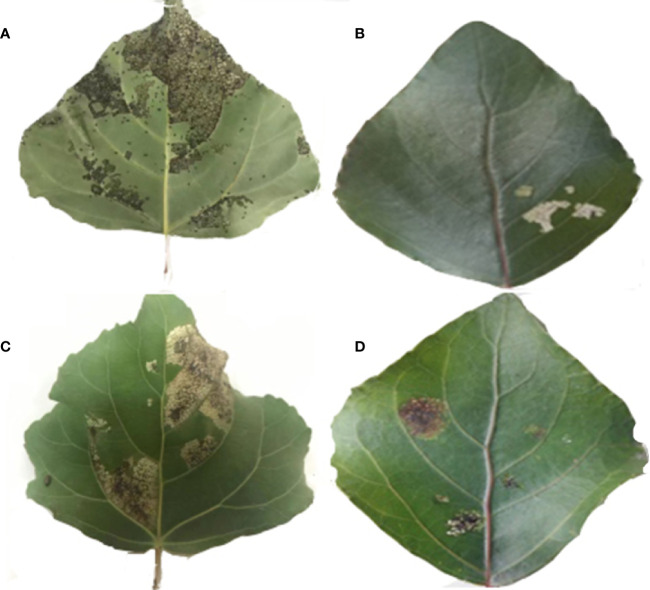
A comparison of leaves from transgenic and non-transgenic lines after 2 days of larval feeding. **(A)** Feeding status of *H. cunea* on non-transgenic leaves. **(B)** Feeding status of *H. cunea* on transgenic leaves. **(C)** Feeding status of *P. versicolora* on non-transgenic leaves. **(D)** Feeding status of *P. versicolora* on transgenic leaves.

Exogenous genes were transformed into *P. nigra* using Agrobacterium leaf-disk transformation. A total of 28 transgenic lines were obtained by kanamycin screening and verified by PCR. Sixteen transgenic lines and the control line that exhibited good growth were selected and fed to first instar larva of *H. cunea* and *P. versicolora*. A comparison of leaves from transgenic and control lines after 2 d of larval feeding is shown in **Figure 2**.

**Figure 3:**
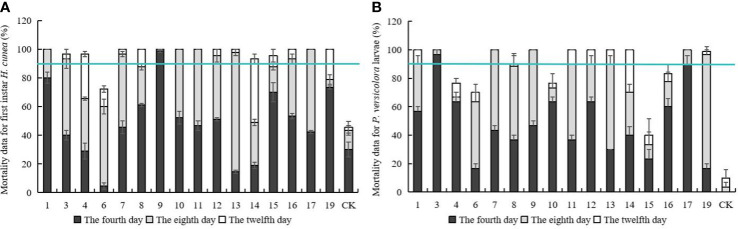
Mortality data for first instar *H. cunea* and Mortality data for *P. versicolora* larvae feeding on transgenic and control plant lines. **(A)** Mortality data for first instar *H. cunea* feeding on transgenic and control plant lines. **(B)** Mortality data for *P. versicolora* larvae feeding on transgenic and control plant lines. 1, 3, 4, 6, 7, 8, 9, 10, 11, 12, 13, 14, 15, 16, 17, and 19 respectively represent the corresponding line numbers of the 16 transgenic poplar lines with good growth; CK, non-transgenic poplar. The number of the first instar h. cunea and p. versicolora larvae bean on transgenic and control plant lines death was counted daily and the mortality rate was calculated. The fourth day, the eighth day, the twelfth day represents the cumulative death rate of four, eight and twelve days. Data are means of three biological replicates of each line. Error bars represent the standard deviation of the mean. The horizontal line is the 90 percent cumulative mortality line.

Mortality data for first instar *H. cunea* and *P. versicolora* larvae feeding on transgenic and control plant lines are shown in **Figure 3**. On the fourth day of feeding, there were significant differences in larval mortality among different lines. On the eighth day, mortality rates began to stabilize, and the mortality rate on some lines reached 100%. In line no. 17, mortality of both insect species was 100% on day 8. On the twelfth day, mortality was higher on all transgenic lines than on controls, and most lines showed significant differences in insect resistance compared with day 8. Five lines with a mortality rate over 90% for both insects on day 8 were identified. (nos. 1, 7, 9, 12, and 17) and used in subsequent tests.

### PCR Detection of Foreign Genes

**Figure 4:**
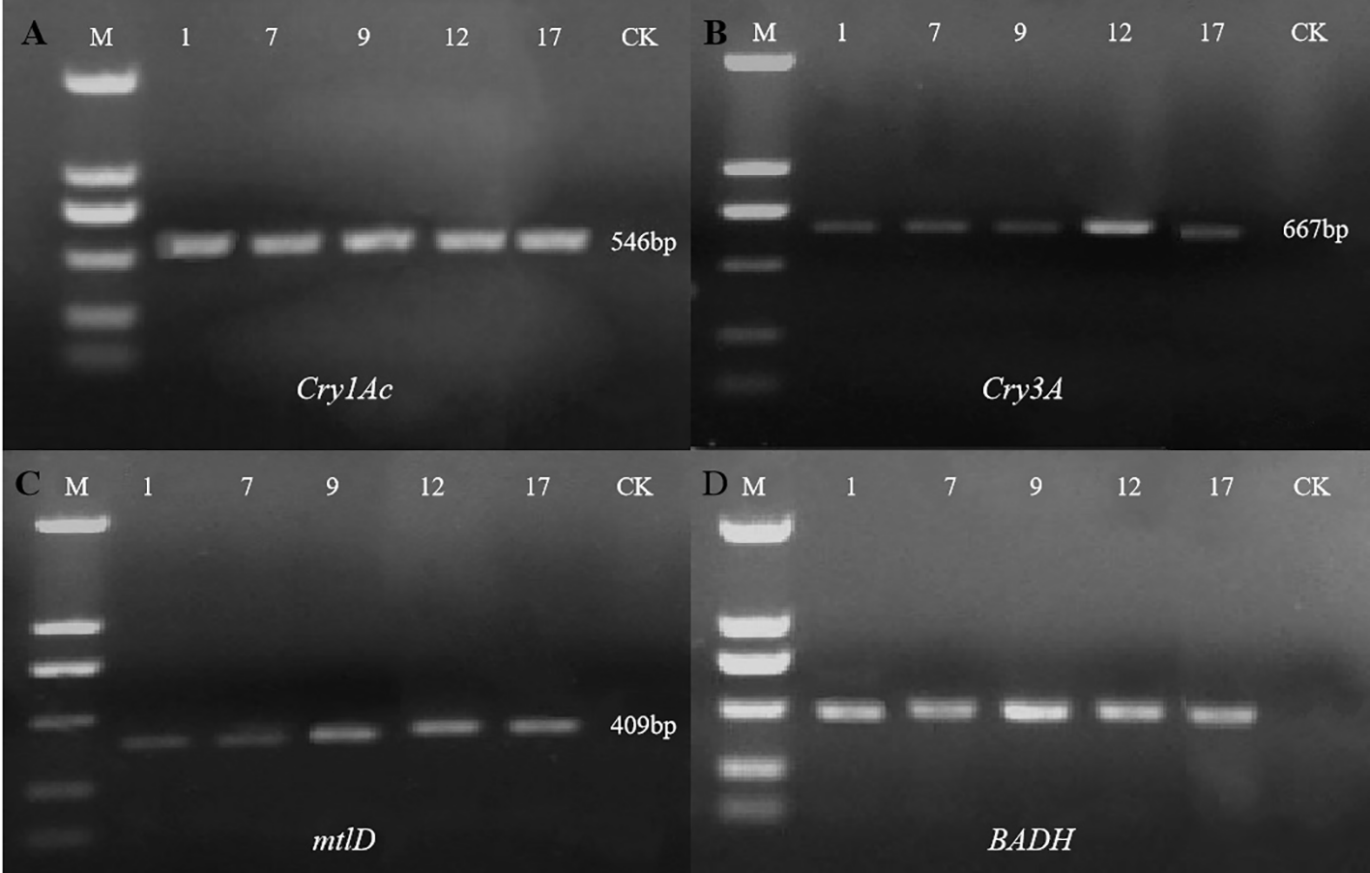
Detection of exogenous genes in five selected transgenic lines. With non-transgenic line (CK) as the control, the exogenous genes in 5 transgenic lines (1, 7, 9, 12, 17) were detected by PCR. M, DL2000 DNA marker (From top to bottom: 2000, 1000, 750, 500, 250, and 100 bp). **(A)** PCR amplification of the Cry1Ac gene; **(B)** PCR amplification of the Cry3A gene; **(C)** PCR amplification of the mtlD gene; **(D)** PCR amplification of the BADH gene.

Agarose gel electrophoresis showed that all four target genes were detected in the five selected transgenic lines but not in the control line (**Figure 4**) (*Cry1Ac*, 546 bp*; Cry3A*, 667 bp; *mtlD,* 409 bp; and *BADH* 507 bp). Preliminary PCR results indicated that all four exogenous genes were successfully introduced into five *P. nigra* eu-1 genomes.

### Fluorescence Quantitative PCR Amplification of Exogenous Genes

Fluorescence signals of the four target genes were detected in all transgenic lines but not in controls. Transcript abundances of the exogenous genes in each line are shown in **Table 3**.

**Table 3:** The transcript abundance of each exogenous gene detected by real-time fluorescence quantitative PCR.

Strain no.	*Cry1Ac*(×10^6^)	*Cry3A*(×10^6^)	*mtlD*(×10^6^)	*BADH*(×10^6^)
1	7.77 ± 1.34^b^	2.13 ± 0.30^cd^	0.6 ± 0.03^d^	5.54± 2.09^c^
7	28.57 ± 5.86^a^	24.23 ± 3.10^a^	77.8 ± 0.36^a^	12.40 ± 1.61^a^
9	2.49 ± 0.24^cd^	23.83 ± 1.43^a^	65.4 ± 0.23^b^	9.50 ± 0.85^b^
12	5.98 ± 0.19^bc^	3.14 ± 0.63^c^	37.4 ± 0.19^c^	6.19 ± 0.22^c^
17	9.92 ± 1.47^b^	14.37 ± 1.33^b^	37.5 ± 0.33^c^	0.09 ± 0.04^d^
CK	0.00 ± 0.00^d^	0.00 ± 0.00^d^	0.00 ± 0.00^e^	0.00 ± 0.00^d^

Data are means of three biological replicates. Use Duncan’s multiple range test, in the same column, the same letter showed that there is no significant difference, the different letters indicate significant difference, p < 0.05.

The transcript abundances of *Cry3A* and *mtlD* were significantly correlated (*p* < 0.01; **Table 4**), but there were no significant correlations between transcript abundances of the other foreign genes.

**Table 4:** Correlation analysis of transcript abundances of *Cry1Ac*, *Cry3A*, *mtlD*, and *BADH* genes.

	*Cry1Ac*	*Cry3A*	*mtlD*	*BADH*
*Cry1Ac*	1			
*Cry3A*	0.554	1		
*mtlD*	0.606	0.919**	1	.
*BADH*	0.598	0.651	0.745	1

**, Significant correlation at 0.01 level.

### Seasonal Variation in Protein Toxin Content in Transgenic Plant Leaves

An ELISA toxin assay was performed on leaves from five selected transgenic lines and controls. Cry1Ac and Cry3A protein toxins were detected in the leaves of transgenic plants at all stages but not in the leaves of control plants. From June to October, Cry1Ac and Cry3A contents were measured monthly in transgenic and control leaves. The content of Cry3A was three orders of magnitude higher than that of Cry1Ac. There were no significant correlations between Bt toxin level and transcript abundance. The Pearson correlation coefficient for *Cry1Ac* was 0.39, and that for *Cry3A* was 0.43.

**Figure 5:**
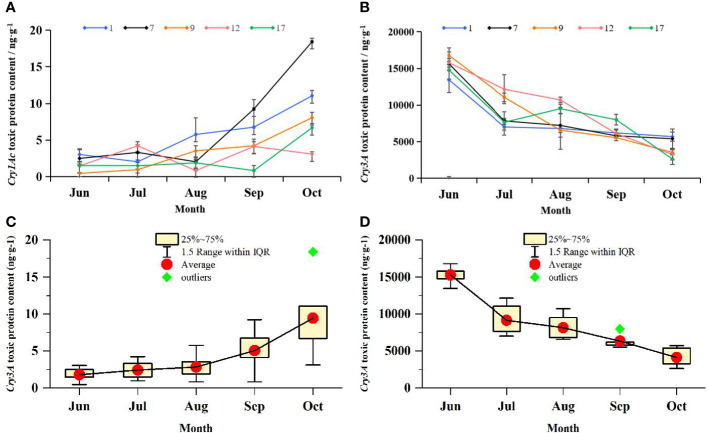
Changes in the content of Cry1Ac and Cry3A protein toxins in transgenic lines over time. ELISA was used to determine the content of toxic protein in transgenic lines. **(A)** Seasonal variation of toxin-protein Cry1Ac content. Data are from the mean values of Cry1Ac toxic protein content in 3 leaves of each transgenic line, three plants were selected for each transgenic line, and one leaf was selected for each plant. **(B)** Seasonal variation of toxin-protein Cry3A content. Data are from the mean values of Cry3A toxic protein content in 3 leaves of each transgenic line, three plants were selected for each transgenic line, and one leaf was selected for each plant. 1, 7, 9, 12, and 17 respectively represent transgenic lines. Line chart shows the change trend of virulence proteins in each line from June to October. Error bars represent the standard deviation of the mean. **(C)** Box plot of seasonal variation of Cry1Ac toxic protein content; Data are from Cry1Ac toxic protein content in all transgenic lines. **(D)** Box plot of seasonal variation of Cry3A toxic protein content; Data are from Cry3A toxic protein content in all transgenic lines.

The Cry1Ac and Cry3A contents and the mean protein toxin contents of transgenic lines varied with the seasons, as shown in **Figure 5**. The Cry1Ac content of most transgenic lines increased gradually from July to October, whereas that of Cry3A decreased gradually over the same time period. The mean Cry1Ac content varied between 3.01 and 9.30 ng·g^−1^. The no. 9 line showed the lowest Cry1Ac content of 0.46 ng·g^−1^ in June, and the no. 7 line showed the highest Cry1Ac content of 18.41 ng·g^−1^ in October. The mean Cry3A content varied among strains from 4110.21 ng·g^−1^ to 15283.842 ng·g^−1^. The no. 9 line showed the highest Cry3A content of 16782.05 ng·g^−1^ in June. In addition to the three orders of magnitude difference between Cry1Ac and Cry3A contents, the two proteins also showed the opposite trend in seasonal variation. The correlation between seasonal changes in Cry1Ac and Cry3A contents was analyzed, and the Pearson correlation coefficient was −0.80. There was therefore a significant negative correlation between the levels of the two proteins (*p* < 0.05), suggesting that the presence of Cry1Ac may have reduced that of Cry3A.

### Resistance of Transgenic Strains to Target Insects of Different Instars

**Figure 6:**
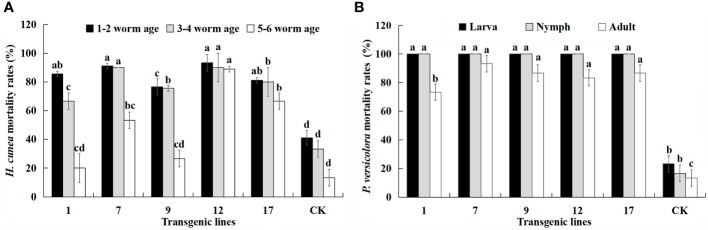
Mortality (%) of all larval ages of *H. cunea* and stages of *P. versicolora* feeding on transgenic lines and control plants. Data are mean of the final mortality rate of larvae of each age in three biological replicates of each line. 1, 7, 9, 12, and 17 correspond to five transgenic lines; CK, non-transgenic line. **(A)** Mortality of all larval ages of *H. cunea* feeding on transgenic lines and control plants. **(B)** Mortality of all stages of *P. versicolora* feeding on transgenic lines and control plants. Bars with different letters indicate significant differences in larval mortality. Error bars represent the standard deviation of the mean. According to Duncan’s multiple range test (*p* < 0.05), different letters indicate significant differences in larval mortality and the same letters indicate no significant differences between the lines.

Multiple instars of *H. cunea* and larvae, nymphs, and adults of *P. versicolora* were used in feeding assays to further characterize the insect resistance of each transgenic line (**Figure 6**). The *H. cunea* resistance assay was performed in July, and the *P. versicolora* assay was performed in September.

The lethality of transgenic lines to *H. cunea* decreased with larval age but was always higher than that of control plants. The mortality of 1–2 instar larvae was relatively stable compared with that of 3–4 instar larvae. The mortality of 5–6 instar larva feeding on transgenic lines was lower, with the exception of the no. 12 line. The mortality of same-aged larvae differed among the transgenic lines. In particular, the Cry1Ac content of the transgenic lines appeared to have a strong effect on *H. cunea* mortality. There was a positive correlation between larval mortality and Cry1Ac content and the correlation coefficients for the three instars were 0.817, 0.774, and 0.721. The positive correlation between mortality rate of 1–2 instar larvae and Cry1Ac content was significant (*p* < 0.05).

The transgenic lines produced higher mortality rates for *P. versicolora* than for *H. cunea*, and the mortality rate of both larvae and nymphs reached 100%. The mortality rate of adult *P. versicolora* was 73–93%, and there were differences among the transgenic lines. There was also a significant positive correlation between insect mortality and Cry3A content (*p* < 0.01). The correlation coefficients for larvae, nymphs, and adults were 0.948, 0.948, and 0.923, respectively. The transgenic lines showed high resistance to *H. cunea* and *P. versicolora*, but insect mortality decreased as insect age increased. The no. 12 and no. 17 lines had the highest and most stable insect resistance.

### Salt Tolerance of Transgenic Lines

**Figure 7:**
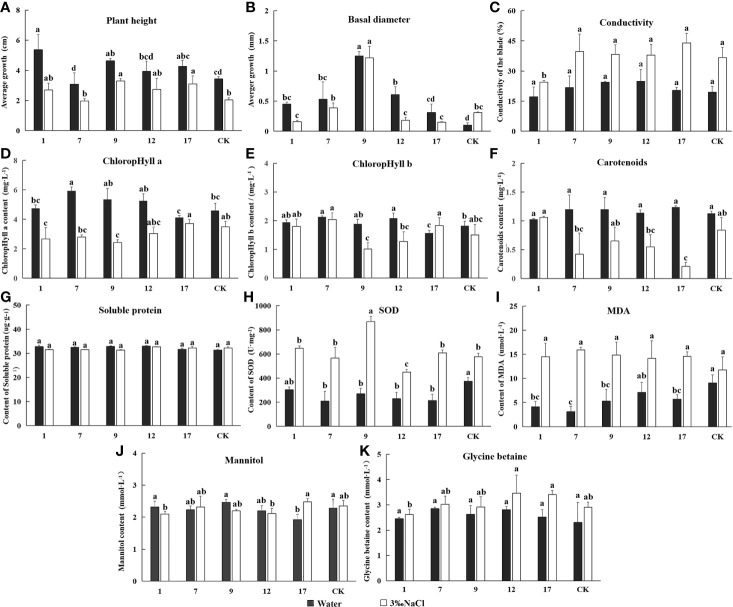
The determination of Eleven indicators related to salt tolerance. Experimentally determined the indexes under the treatment of water and 3‰ NaCl concentration. In the diagram, gray represents water treatment and white represents 3‰ NaCl treatment. 1, 7, 9, 12, and 17 stands for five transgenic lines; CK, non-transgenic line. Data are means of three biological replicates of each line, error bars represent the standard deviation of the mean. According to Duncan’s multiple range test (*p* < 0.05), different letters indicate significant differences and the same letters indicate no significant differences between the lines. **(A)** Bar chart of plant height determination results. **(B)** Bar chart of ground diameter determination results. **(C)** Bar chart of leaf conductivity determination results. **(D)** Bar chart of ground diameter determination results. **(E)** Bar chart of contents chlorophyll a determination results. **(E)** Bar chart of contents chlorophyll b determination results. **(F)** Bar chart of contents carotenoids determination results. **(G)** Bar chart of superoxide dismutase (SOD) activity determination results. **(H)** Bar chart of malondialdehyde (MDA) activity determination results. **(I)** Bar chart of soluble protein content determination results. **(J)** Bar chart of mannitol content determination results. **(K)** Bar chart of glycine betaine content determination results.

We measured plant height, basal diameter, relative conductivity, chlorophyll content, and contents mannitol and glycine betaine in transgenic lines and controls treated with water and 3‰ NaCl for 20 d. All results are shown in **Figure 7**. Plant height growth and basal diameter decreased under salt treatment, with the exception of basal diameter in control lines, indicating that plant growth was generally inhibited by salt stress. Leaf conductivity of all lines increased significantly under salt stress, indicating a loss of cytoplasmic electrolytes and a higher degree of cellular damage particularly to cell membranes. The chlorophyll content of plant leaves gradually decreased under salt stress, a result that would have directly affected the light reaction apparatus and all plants in reduced photosynthetic capacity. The activity of SOD and MDA also increased significantly in response to salt. SOD is mainly used to remove reactive oxygen radicals from plant cells and is therefore an important protective enzyme (Williamson and Richardson, 1988). MDA content is associated with stress-induced damage and causes cytotoxicity by reacting with various cellular components. Lower MDA content is associated with better plant stress resistance (Liu et al., 2003). In this experiment, MDA content increased equally in transgenic and control lines. This result suggests, a variety of enzymes and membrane systems may have been damaged under salt stress, and transgenic plants did not differ in this regard. Soluble protein content was similar in all lines, and there were no differences in mannitol and glycine betaine between transgenic and control plants under normal or salt stress conditions, with the exception of the no. 17 line. The presence of *mtlD* and *BADH* genes therefore had little impact on plant salt tolerance.

**Figure 8:**
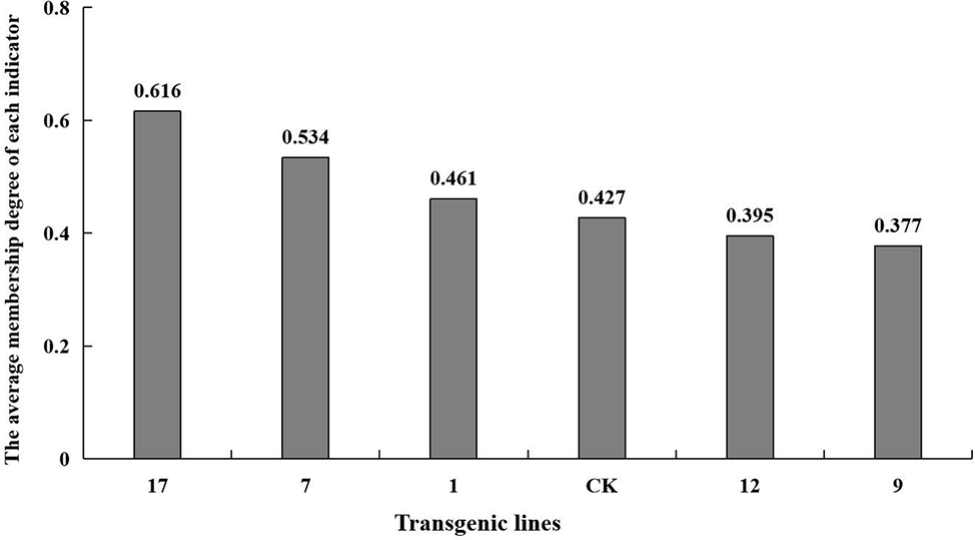
The average membership degree of each indicator. The average membership degree of transgenic lines (1, 7, 9, 12, 17) and non-transgenic lines (CK) are listed from highest to lowest.

The fuzzy mathematical membership function method was used to quantitatively transform the eleven indicators, and the average membership degree of each indicator. The average membership degree of each line was used as the comprehensive identification standard to compare salt tolerance among the lines. The salt tolerance of the plant lines was ranked as shown in **Figure 8**. Transgenic lines differed in their degree of salt tolerance, and line no. 17 had the highest salt tolerance with a mean membership degree of 0.61. The mean membership degree of the non-transgenic control was 0.43, and the salt tolerance indices of transgenic lines no. 9 and no. 12 were lower than that of the control group.

**Figure 9:**
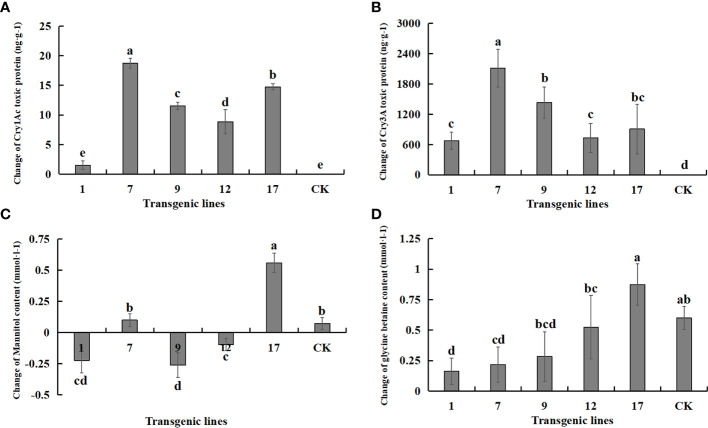
Changes in the content of Insect-resistant gene products and salt-tolerant gene pathway products under salt stress. Insect-resistant gene products: Cry1Ac and Cry3A protein toxins; salt-tolerant gene pathway products: mannitol and glycine betaine. Data are means of three biological replicates of each line, error bars represent the standard deviation of the mean, which is obtained by subtracting the content of each substance in water treatment from the content of each substance in 3‰ NaCl concentration. According to Duncan’s multiple range test (*p* < 0.05), different letters indicate significant differences and the same letters indicate no significant differences between the lines. **(A)** Changes of Cry1Ac protein toxin content. **(B)** Changes of Cry3A protein toxin content. **(C)** Changes of mannitol content. **(D)** Changes of glycine betaine content.

The expression of each exogenous gene under salt stress and control conditions is shown in **Figure 9**. The content of both protein toxins increased significantly, suggesting that the transgenic lines had excellent potential resistance to insects. The content of betaine and mannitol, which contribute to the improvement of plant salt tolerance, did not increase significantly under salt stress. The two salt-tolerance genes were not effectively expressed under salt stress, and the content of betaine and mannitol was not improved compared to the non-transgenic control. In fact, the content of betaine and mannitol decreased in some transgenic lines.

## Discussion

**Figure 10:**
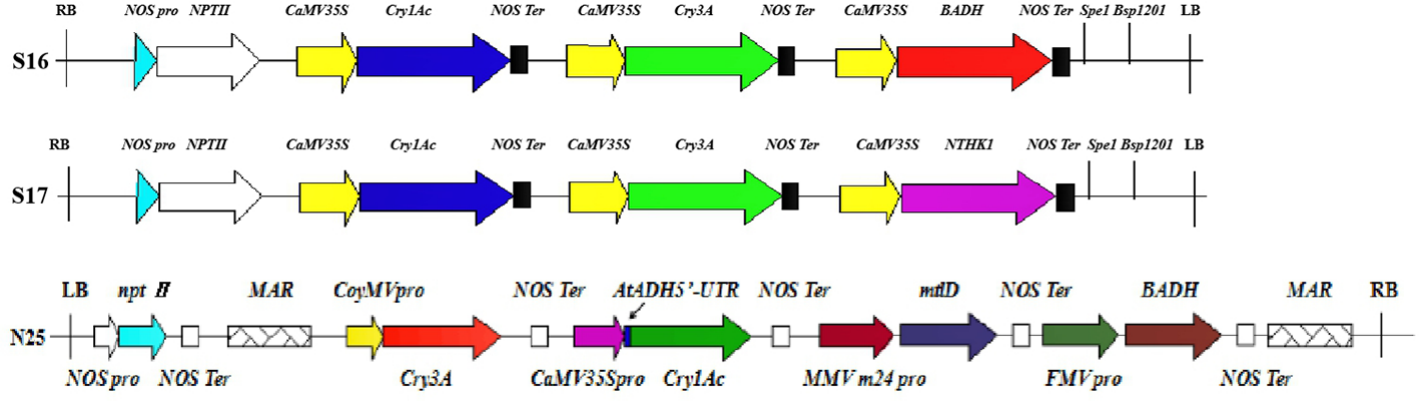
Comparison of multigene carrier structures. S16, S17, and N25 represent the carrier structures of Yang et al. (2016) and Liu et al. (2016), and this experiment. Different colors and shapes represent different components. RB, right boundary of vector. NOS pro, promoter of neomycin phosphotransferase gene; *nptII*, neomycin phosphotransferase gene; CaMV35S, cauliflower mosaic virus CaMV35S promoter; *Cry1Ac*, bacillus thuringiensis insecticidal crystal protein gene; NOS Ter, terminator of neomycin phosphotransferase gene; *Cry3A*, bacillus thuringiensis insecticidal crystal protein gene; *BADH*, betaine aldehyde dehydrogenase gene; Spe1 and Bsp1201, restriction enzyme cutting site; LB, vector left boundary; *NTHK1*, an ethylene receptor gene cloned from tobacco; MAR, the molecular structure of the matrix attachment region of tobacco; CoyMV pro, commelina yellow mottle virus promoter; AtADH5’-UTR, translation enhancer; MMV m24 pro, mirabilis mosaic virus promoter; *mtlD*, 1-phosphomannitol dehydrogenase gene; FMV pro, figwort mosaic virus promoter.

Plant transformation with multiple genes is an emerging trend in plant genetic engineering promising to improve plant traits through the efficient expression of multiple exogenous genes (Liu et al., 2011). Single gene transfer can change only one trait, but multi-gene transformation can improve many plant traits simultaneously with precision and efficiency (Wang et al., 2016). Studies have shown that poplar with the *Cry1Ac* gene has high resistance to *Lepidoptera* such as *H. cunea, Clostera anachoreta,* and *Lymantria dispar*. Likewise, poplar with the *Cry3A* gene is lethal to *Coleoptera*, producing close to 100% mortality in first instar larvae (Yang et al., 2003; Wang Y.P. et al., 2008; Niu et al., 2011). The two *Bt* genes have been inserted into a single vector to produce resistance to both *Lepidoptera* and *Coleoptera* pests. However, previous studies have suggested that the two *Bt* genes cannot be expressed efficiently and simultaneously in poplar using multigene expression vectors (Yang et al., 2006; Wang et al., 2012; Dong et al., 2015; Zhao et al., 2016; Wang et al., 2018). Yang et al. (2016) constructed a vector with two *Bt* genes and the salt tolerance gene *BADH* and used it to transform *Populus euramericana* 107. The transgenic strains had a high mortality rate for *Coleoptera*, only the highest mortality rate for *Lepidopteran H. cunea* larvae was only 66.7%. Liu et al. (2016) constructed a vector with two *Bt* genes and the salt tolerance gene *NTHK1* and used it to transform *P. euramericana* 107. The transgenic strains also had a high mortality rate for *Coleoptera* but not for *Lepidoptera*. **Figure 10**. presents a comparison of the multigene vector construct used in the current work with those of Yang et al. (2016) and Liu et al. (2016).

A comparison of the insect resistance conferred by different multigene vector structures and transgenic lines is presented in **Table 5**. Neither the transgenic strains of Yang et al. (2016) nor those of Liu et al. (2016) exhibited high salt tolerance.

**Table 5:** Comparison of the insect resistance conferred by different multigene vector structures and transgenic lines.

The carrier	S16	S17	N25
Receptor species	*Populus euramericana 107*	*Populus euramericana 107*	European *Populus nigra L*
Number of foreign genes	3	3	4
Promoter	yes	yes	yes
Enhancer	not	not	AtADH 5′-UTR
MAR	not	not	yes
Close to the NptII gene	*Cry1Ac*	*Cry1Ac*	*Cry3A*
Mortality of 1-age *Lepidoptera* larvae	42.2%∼66.7%	0.0%∼68.89%	77%–97%
Mortality of 1-year-old *Coleoptera* larvae	100%	100%	100%

S16, S17, and N25 represent the carrier structures of Yang et al. (2016); Liu et al. (2016) and this experiment. The comparison data are from the above two references and the statistical data of this experiment.

In the present experiment, two *Bt* genes and two salt-tolerance genes were inserted into the same plant expression vector, along with additional components selected to enhance target gene expression. Using the same promoter for different exogenous genes can trigger gene silencing. To avoid this issue, we selected four different promoters for the four exogeneous genes: CaMV35S (cauliflower mosaic virus promoter), CoYMV (commelina yellow mottle virus), FMV (figwort mosaic virus), and MMV (mirabilis mosaic virus). The CaMV35S promoter is a constitutive promoter capable of initiating the expression of exogenous genes in most plants; it is the most widely used promoter in genetic engineering (Holtorf et al., 1995; Wilmink et al., 1995; Mitsuhara et al., 1996). CoYMV has shown even greater activity than CaMV35S in tobacco and maize suspension cells (Medberry et al., 1992), and the CaMV35S and FMV promoters share similar expression patterns and activities in tobacco protoplasts (Sanger et al., 1990; Fits and Memelink, 1997). MMV promoter fragments also have strong promoter activity, especially in monocots (Dey and Maiti, 1999). Nonetheless, studies indicate that the same promoter may have different initiation strengths in different plants (Zhang et al., 2004). Many strong promoters can substitute for the 35S promoter in monocots, but promoters other than 35S are relatively weakly expressed in dicots (Schledzewski and Mendel, 1994; Yuan et al., 2000; Zhang et al., 2005; Nie et al., 2008). Because the intensity of the other promoters was expected to be weaker than that of 35S in poplar, we added MAR structures to both ends of the vector to reduce the effect of the plant’s own regulatory mechanisms on target gene expression (Luo et al., 2017). The expression of Cry1Ac may not be ideal when the two Bt genes coexist, and their mutual expression inhibition may be related to their position and direction in the vector (Dong et al., 2015; Qiu et al., 2017; Zhang et al., 2019). Against this background, the AtADH5’-UTR translation enhancer was added before the *Cry1Ac* gene, and the position of the two Bt genes in the vector was adjusted to improve *Cry1Ac* expression and *Lepidoptera* resistance. The five most effective transgenic lines had a mortality rate greater than 80% for all ages of *Lepidoptera* larvae and *Coleoptera* insects sensitive to Cry1Ac. Both Bt genes were highly expressed, and the transgenic plants showed excellent double insect resistance. On the other hand, no transgenic strains with high salt tolerance were obtained.

The expression ability of exogenous insect-resistance genes in transgenic plants determines their insecticidal effect (Ren et al., 2017; Xiao et al., 2017). Accurate analysis of protein toxin expression conferred by exogenous genes is very important for the identification and screening of transgenic lines with high insect resistance. In this experiment, compared with previous studies (Zhang et al., 2016; Zhang et al., 2019), the two Bt protein toxins showed different patterns of seasonal variation. This seasonal change in protein content may be due to its accumulation in the plant, similar seasonal changes have been documented in protein expression and other metabolic processes, this change related to the intrinsic metabolism of the plant itself (Shen et al., 2010). The expression of exogenous genes is related to the development of plant species. With the increase of tree growth age, the seasonal variation of Bt toxin protein needs to be further verified. The exogenous genes showed no significant differences at the transcriptional level. However, there are significant differences in the level of translation. Levels of Cry3A protein were three orders of magnitude higher than those of Cry1Ac protein. *Cry3A* gene has a greater GC content than the partially modified *Cry1Ac* gene, its GC content is closer to that of plants, which is conducive to its stable expression in transgenic lines (Wang et al., 2012; Liu et al., 2016). Although the expression level of *Cry3A* was affected by its promoter, the expression level of the gene itself was higher, and the content of toxic protein was higher, thus failing to affect its insect resistance. Expression of the *Cry1Ac* gene was not only driven by the 35S strong promoter, but was also improved by the presence of enhancer elements. The expression of the salt-tolerance genes in the transgenic lines were not ideal, and the salt tolerance of the lines was not strong. The low strength of the promoters used for the salt-tolerance genes help to explain the poor salt tolerance of the transgenic lines. In the process of translation, the insect-resistant genes and the salt-tolerant genes compete with each other for photosynthate (Winicov and Seemann, 1990), and the salt-tolerance genes may be inhibited due to their inferior position, resulting in poor salt tolerance (Jiang et al., 2003; Si et al., 2007; Wang et al., 2018). Under salt stress, poplar undergoes a series of physiological and biochemical responses that affect photosynthesis and respiration (Zhou et al., 2004; Zhou, 2006; Luo et al., 2017). Mechanisms of plant salt tolerance are quite complex, and only increasing the expression of two exogenous genes may not have been sufficient to improve salt tolerance (Zong and Yang, 2011).

To obtain transgenic plants with various desirable traits and improve the expression of exogenous genes, the expression and interaction mechanisms of exogenous genes after multi-gene transformation will require further study. Here, the construction characteristics of the vector clearly promoted the expression of the exogenous genes. The selection of suitable promoters and the optimization of their activity is a primary consideration for enhancing the expression of exogenous genes. Currently, constitutive promoters are widely used in plant expression vectors, and the use of natural promoters often fails to generate satisfactory results (Jiao et al., 2019). The modification of existing promoters and the construction of compound promoters will therefore be very important means for efficient expression of exogenous genes. Many factors can explain transcriptional differences (Cervera et al., 2000; Chandler and Vaucheret, 2001) such as differences in the genes themselves, insertion sites of the exogenous genes, copy numbers of exogenous genes, sequences of exogenous genes, and interference interactions among genes. The MAR structure can effectively reduce the differences in transcription levels caused by different promoters, but it cannot eliminate gene silencing at the post-transcriptional level (Anandalakshmi et al., 1998). In subsequent experiments, translation enhancers can be added before salt-tolerance genes to improve their expression. Reducing gene silencing at the translation level will be the goal of future experiments. How to obtain transgenic plants with desired traits, coordinate the expression levels of transcription and translation, and reduce the unpredictability of gene expression are important questions to address in genetic engineering.

## Data Availability Statement

The raw data supporting the conclusions of this article will be made available by the authors, without undue reservation, to any qualified researcher.

## Author Contributions

XZ and YD conceived the study. XZ analyzed the data and edited the manuscript. QZ and DX collect data and analyzed the data. MY and JW designed the experiments and revised the manuscript.

## Funding

This study was supported by the National Key Program on Transgenic Research (2018ZX08020002) and the Basic Research Plan Project of Hebei Province (18966801D).

## Conflict of Interest****

The authors declare that the research was conducted in the absence of any commercial or financial relationships that could be construed as a potential conflict of interest.
