# Corrigendum: Expression of Multiple Exogenous Insect Resistance and Salt Tolerance Genes in *Populus nigra* L.

**DOI:** 10.3389/fpls.2021.661655

**Published:** 2021-03-08

**Authors:** Xinglu Zhou, Yan Dong, Qi Zhang, Dandan Xiao, Minsheng Yang, Jinmao Wang

**Affiliations:** ^1^Institute of Forest Biotechnology, Forestry College, Agricultural University of Hebei, Baoding, China; ^2^Hebei Key Laboratory for Tree Genetic Resources and Forest Protection, Baoding, China; ^3^Institute of Coastal Agriculture, Hebei Academy of Agriculture and Forestry Sciences, Shijiazhuang, China

**Keywords:** multi-resistance gene, multigenic vector, *Populus nigra* L., insect resistance, salt resistance

In the original article, there were four mistakes as published, respectively, letters (a, b, c, d,…) for Duncan's multiple range test was assigned wrongly in Table 3, Figure 6, Figure 7 and Figure 9. In Table 3, transcriptional abundance superscript the letters were assigned wrongly. In Figure 6, the letters above the mortality rate were assigned wrongly. In Figure 7, the letters above the content of each indicator were assigned wrongly. In Figure 9, the letters above the change in products were assigned wrongly.

**Table 3:** The transcript abundance of each exogenous gene detected by real-time fluorescence quantitative PCR.

**Strain no.**	***Cry1Ac*(×10^**6**^)**	***Cry3A*(×10^**6)**^**	***mtlD*(×10^**6**^)**	***BADH*(×10^**6**^)**
1	7.77 ± 1.34^b^	2.13 ± 0.30^cd^	0.6 ± 0.03^d^	5.54± 2.09^c^
7	28.57 ± 5.86^a^	24.23 ± 3.10^a^	77.8 ± 0.36^a^	12.40 ± 1.61^a^
9	2.49 ± 0.24^cd^	23.83 ± 1.43^a^	65.4 ± 0.23^b^	9.50 ± 0.85^b^
12	5.98 ± 0.19^bc^	3.14 ± 0.63^c^	37.4 ± 0.19^c^	6.19 ± 0.22^c^
17	9.92 ± 1.47^b^	14.37 ± 1.33^b^	37.5 ± 0.33^c^	0.09 ± 0.04^d^
CK	0.00 ± 0.00^d^	0.00 ± 0.00^d^	0.00 ± 0.00^e^	0.00 ± 0.00^d^

*Data are means of three biological replicates. Use Duncan's multiple range test, in the same column, the same letter showed that there is no significant difference, the different letters indicate significant difference, P < 0.05*.

**Figure 6:**
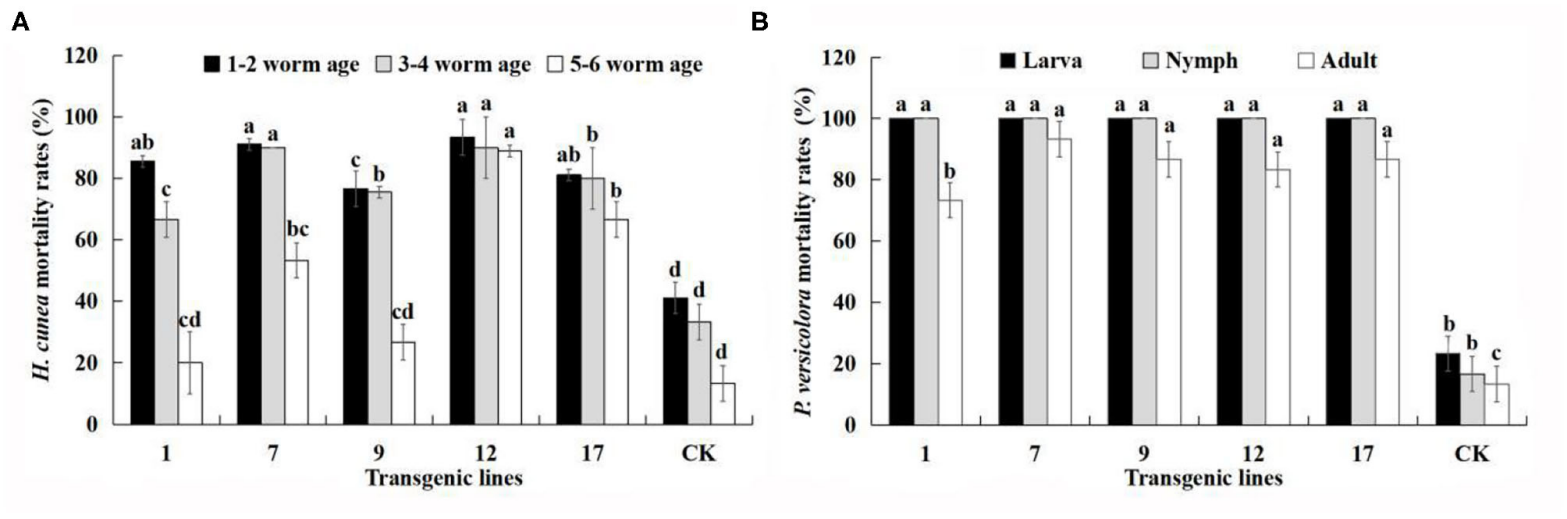
Mortality (%) of all larval ages of *H. cunea* and stages of *P. versicolora* feeding on transgenic lines and control plants. Data are mean of the final mortality rate of larvae of each age in three biological replicates of each line. 1, 7, 9, 12, and 17 correspond to five transgenic lines; CK, non-transgenic line. **(A)** Mortality of all larval ages of *H. cunea* feeding on transgenic lines and control plants. **(B)** Mortality of all stages of *P. versicolora* feeding on transgenic lines and control plants. Bars with different letters indicate significant differences in larval mortality. Error bars represent the standard deviation of the mean. According to Duncan's multiple range test (*p* < 0.05), different letters indicate significant differences in larval mortality and the same letters indicate no significant differences between the lines.

**Figure 7:**
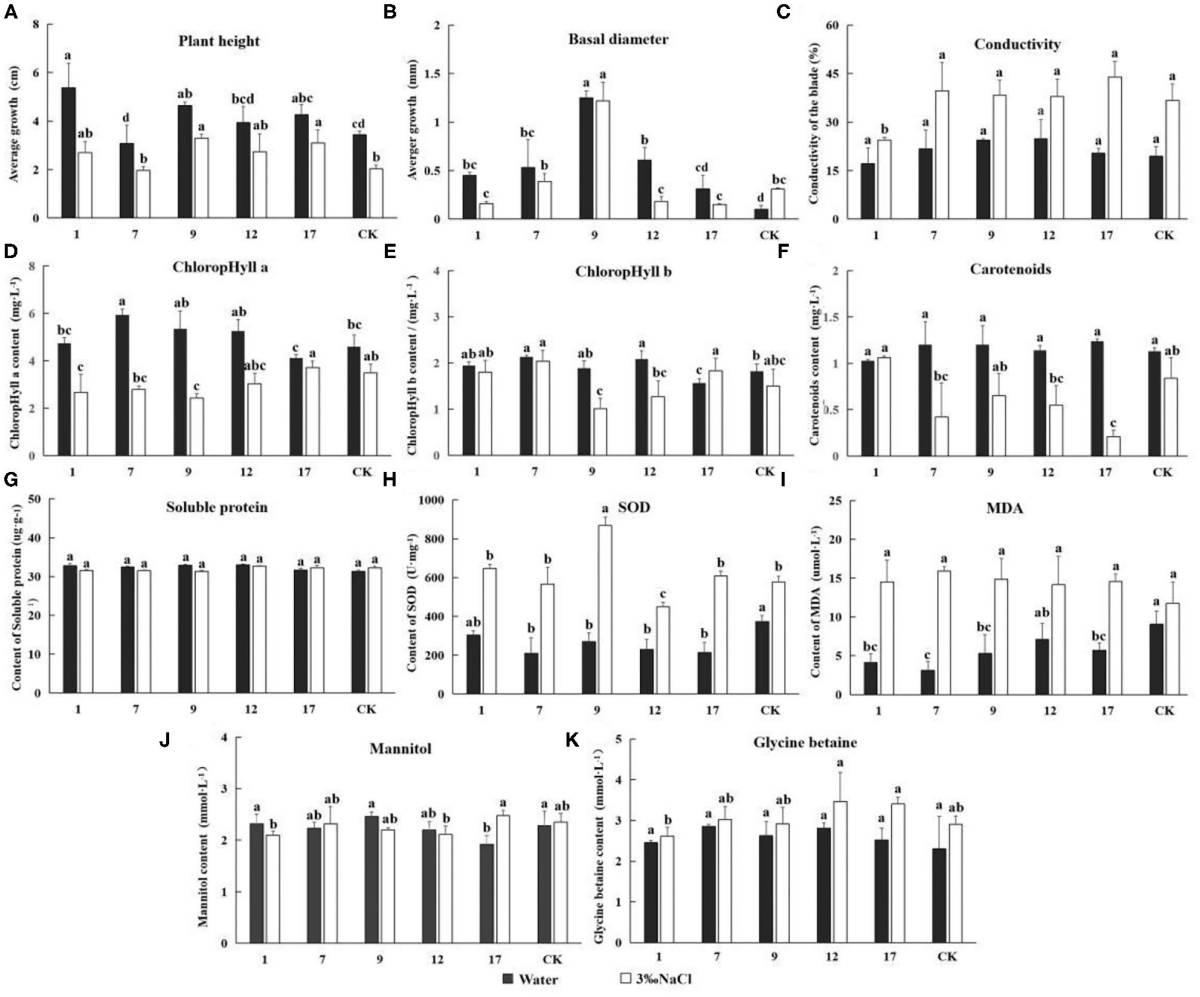
The determination of Eleven indicators related to salt tolerance. Experimentally determined the indexes under the treatment of water and 3‰ NaCl concentration. In the diagram, gray represents water treatment and white represents 3‰ NaCl treatment. 1, 7, 9, 12, and 17 stands for five transgenic lines; CK, non-transgenic line. Data are means of three biological replicates of each line, error bars represent the standard deviation of the mean. According to Duncan's multiple range test (*p* < 0.05), different letters indicate significant differences and the same letters indicate no significant differences between the lines. **(A)** Bar chart of plant height determination results. **(B)** Bar chart of ground diameter determination results. **(C)** Bar chart of leaf conductivity determination results. **(D)** Bar chart of ground diameter determination results. **(E)** Bar chart of contents chlorophyll a determination results. **(E)** Bar chart of contents chlorophyll b determination results. **(F)** Bar chart of contents carotenoids determination results. **(G)** Bar chart of superoxide dismutase (SOD) activity determination results. **(H)** Bar chart of malondialdehyde (MDA) activity determination results. **(I)** Bar chart of soluble protein content determination results. **(J)** Bar chart of mannitol content determination results. **(K)** Bar chart of glycine betaine content determination results.

**Figure 9:**
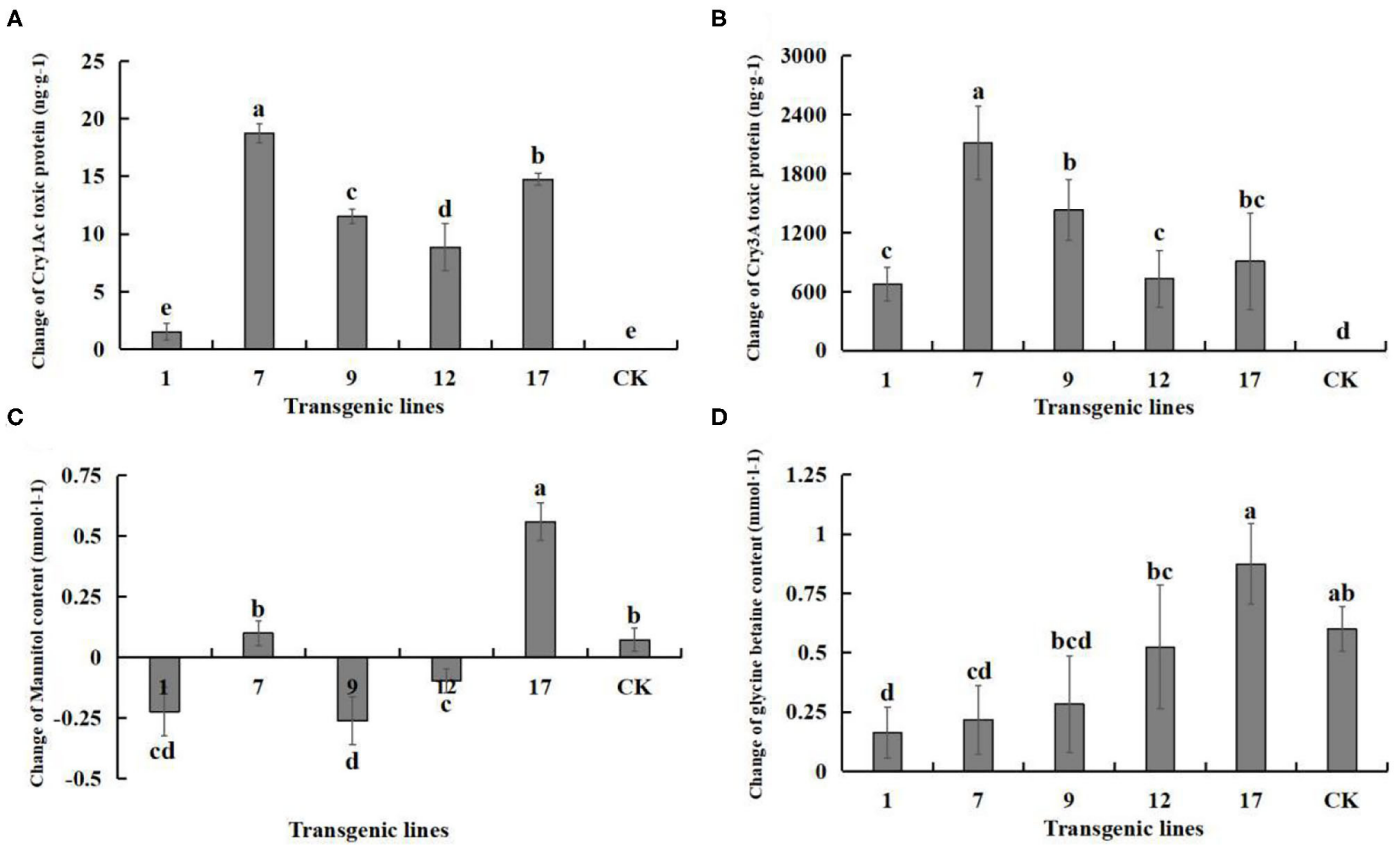
Changes in the content of Insect-resistant gene products and salt-tolerant gene pathway products under salt stress. Insect-resistant gene products: Cry1Ac and Cry3A protein toxins; salt-tolerant gene pathway products: mannitol and glycine betaine. Data are means of three biological replicates of each line, error bars represent the standard deviation of the mean, which is obtained by subtracting the content of each substance in water treatment from the content of each substance in 3‰ NaCl concentration. According to Duncan's multiple range test (*p* < 0.05), different letters indicate significant differences and the same letters indicate no significant differences between the lines. **(A)** Changes of Cry1Ac protein toxin content. **(B)** Changes of Cry3A protein toxin content. **(C)** Changes of mannitol content. **(D)** Changes of glycine betaine content.

The corrected Table 3 appears below.

The corrected Figure 6 appears below.

The corrected Figure 7 appears below.

The corrected Figure 9 appears below.

We are deeply sorry for the trouble our mistake has caused readers. There is no effect on the paper except for the error in the letter marking of the original article.

The authors apologize for this error and state that this does not change the scientific conclusions of the article in any way. The original article has been updated.

